# Preoperative and Postoperative Optical Coherence Tomography Findings in Patients with Rhegmatogenous Retinal Detachment Involving the Macular Region

**DOI:** 10.1155/2013/426867

**Published:** 2013-09-11

**Authors:** Asaki Matsui, Hiroshi Toshida, Rio Honda, Takahiko Seto, Toshihiko Ohta, Akira Murakami

**Affiliations:** ^1^Department of Ophthalmology, Juntendo University Shizuoka Hospital, 1129 Nagaoka, Izunokuni, Shizuoka 410-2295, Japan; ^2^Department of Ophthalmology, Juntendo University School of Medicine, 2-1-1 Hongo, Bunkyo, Tokyo 113-8421, Japan

## Abstract

*Purpose*. To evaluate morphologic changes of the macula, we observed eyes with rhegmatogenous retinal detachment (RRD) involving the macular region by optical coherence tomography (OCT). *Subjects and Methods*. We studied 26 eyes with RRD before and after surgery, assessing visual acuity, the height of retinal detachment at the fovea (HRD), and morphologic changes of the macular region. The interval between the onset and surgery was also determined. We examined the external limiting membrane (ELM) after surgery and the continuity of the inner segment-outer segment junction (IS/OS junction) of the photoreceptor layer. *Results*. Impairment of visual acuity was observed when HRD was over 1,000 **μ**m, when there was outer nuclear layer edema before surgery, and when there was IS/OS junction disruption 3 months after surgery. However, 67% of eyes with a continuous ELM and IS/OS junction disruption 3 months after surgery eventually showed restoration of the continuity of IS/OS junction at 6 months. *Conclusions*. Impairment of visual acuity was observed in eyes with HRD >1,000 **μ**m, preoperative outer nuclear layer edema, and IS/OS junction disruption 3 months postoperatively. It is suggested that continuity of ELM might affect restoration of IS/OS junction after surgery for retinal detachment.

## 1. Introduction

Surgery is required to reattach the retina in most eyes with rhegmatogenous retinal detachment (RRD). If the interval from onset to surgery is too long or if reattachment cannot be achieved, serious visual impairment can result, with blindness occurring in the worst cases. Various treatments for RRD are available depending on its severity and progression. Vitrectomy is sometimes required, especially for RRD involving the macular region (macula-off RRD). However, even when reattachment of the retina is achieved, postoperative visual impairment is generally unavoidable, and the visual outcome varies from case to case.

 In recent years, the structure of the retina has been clarified owing to advances in optical coherence tomography (OCT), and various reports have been published concerning factors that determine the visual outcome after retinopexy in patients with RRD [1–6]. In this study, determinants of the visual outcome were investigated retrospectively by spectral domain OCT in eyes with macula-off RRD that underwent surgical reattachment of the retinal.

## 2. Subjects and Methods

The subjects were 26 patients (26 eyes) who underwent surgery after the diagnosis of macula-off RRD at the Department of Ophthalmology of Juntendo University, Shizuoka Hospital between April 2010 and November 2011. In these patients, reattachment of the retina was achieved by initial surgery, and postoperative followup was continued for at least 6 months. Patients with other macular diseases or with the complication of proliferative vitreoretinopathy were excluded from this study. Vitrectomy was performed in 19 patients (19 eyes), while scleral buckling was done in 7 patients (7 eyes). The day of onset was defined as the day when the patient became aware of a visual field defect or visual impairment.

The patients were examined to determine the preoperative and postoperative visual acuity. Visual acuity data was done after transformation; the data were converted to logarithm of the minimum angle of resolution (logMAR) values. Preoperative HRD was measured by HD-OCT (Cirrus: manufactured by Zeiss), preoperative retinal architecture, and postoperative ELM and IS/OS junction continuity.Based on the data obtained by these examinations, the following 6 parameters were investigated:

1. relationship between the duration of retinal detachment and postoperative visual acuity,
2. relationship between the preoperative HRD and preoperative/postoperative visual acuity,
3. relationship between the time until the resolution of retinal detachment at the fovea (HRD = 0) and postoperative visual acuity,
4. investigation of the preoperative structure of the retina and comparison between eyes with a normal retinal architecture and eyes with edema of the outer nuclear layer,
5. relationship of postoperative visual acuity to postoperative IS/OS junction continuity,
6. relationship of postoperative visual acuity to postoperative IS/OS junction and ELM continuity.

Statistical analysis was performed by the Mann-Whitney *U* test, and differences of *P* < 0.05 were considered significant.

## 3. Results

The subjects were 19 men (19 eyes) and 7 women (7 eyes) with a mean age of 50.7 ± 15.6 years (range: 28 to 72 years). The mean follow-up period was 11 months (range: 6 to 28 months). The mean logMAR visual acuity was 0.66 ± 0.67 before surgery and 0.32 ± 0.42 6 months after surgery.

### 3.1. Relationship between the Duration of Retinal Detachment and Postoperative Visual Acuity

The mean interval from onset to surgery was 23.2 days (range: 3 to 120 days). The logMAR visual acuity 6 months after surgery was 0.27 ± 0.38 for 12 patients in whom this interval was ±10 days and 0.45 ± 0.49 for 14 patients in whom this interval was ±11 days. The visual outcome showed no significant difference in relation to the interval from onset to surgery.

### 3.2. Relationship between Preoperative HRD and Preoperative/Postoperative Visual Acuity

**Figure 1:**
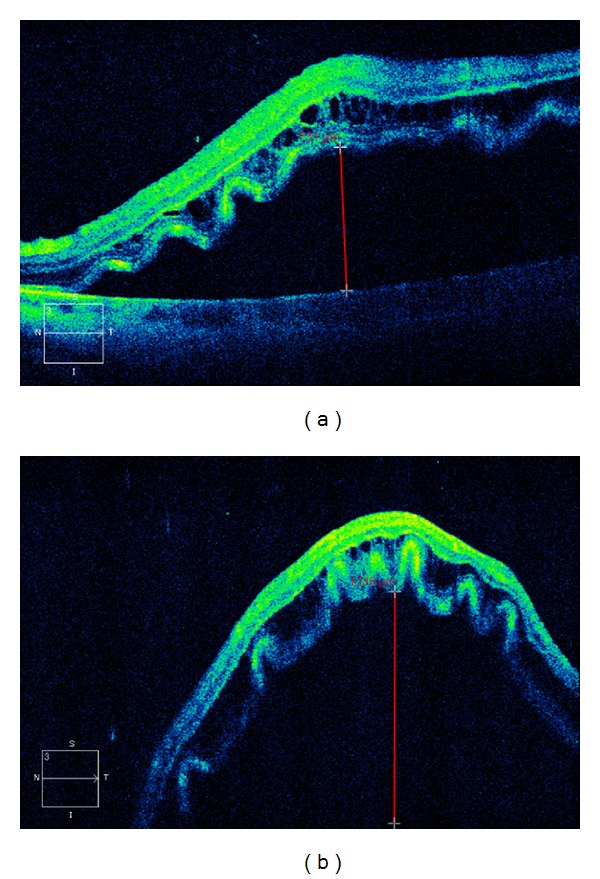
Preoperative optical coherence tomography (OCT) findings for the eyes with height of retinal detachment (HRD). (a) A 70-year-old male with preoperative HRD < 1000 *μ*m in his left eye. The preoperative logMAR visual acuity was 0.39, and the duration of retinal detachment was 60 days. (b) A 49-year-old female with preoperative HRD ≧ 1000 *μ*m in her left eye. Her preoperative logMAR visual acuity was 2.00, and the duration of retinal detachment was 30 days.

The mean preoperative HRD of all cases was 561 ± 521 *μ*m (range: 76 to 1,704 *μ*m).  Table 1 shows the relationship between preoperative HRD and visual acuity. In 7 eyes with a preoperative HRD ≥ 1,000 *μ*m, the mean logMAR visual acuity was 1.54 ± 0.50 before surgery and 0.70 ± 0.36 after surgery. In 19 eyes with an HRD < 1,000 *μ*m, the mean logMAR visual acuity was 0.65 ± 0.67 before surgery and 0.25 ± 0.38 after surgery. The mean preoperative visual acuity and the visual acuity 6 months after surgery showed a significant difference between the HRD ≥ 1,000 *μ*m and HRD < 1,000 *μ*m groups (*P* < 0.05, Mann-Whitney *U*-test). There were no significant differences in the mean age between HRD ≥ 1,000 *μ*m and HRD < 1,000 *μ*m groups.  Figure 1 shows the typical OCT findings.

### 3.3. Relationship between the Time until the Resolution of Foveal Detachment and Postoperative Visual Acuity

The mean logMAR visual acuity 6 months postoperatively was 0.12 ± 0.23 for 16 eyes (vitreous surgery: 14 eyes, scleral buckling: 2 eyes) in which foveal detachment was resolved within 3 months after surgery, while it was 0.51 ± 0.48 for 10 eyes (vitreous surgery: 4 eyes, scleral buckling: 6 eye) in which detachment persisted for 3 months and more. The visual acuity 6 months postoperatively was significantly better when foveal detachment resolved within 3 months after surgery than when it persisted for 3 months or more after surgery (*P* < 0.01). Foveal detachment persisted for 3 months or more in 4 eyes (25%) after vitrectomy and in 6 eyes (75%) after scleral buckling.

### 3.4. Influence of Preoperative Retinal Architecture

**Figure 2:**
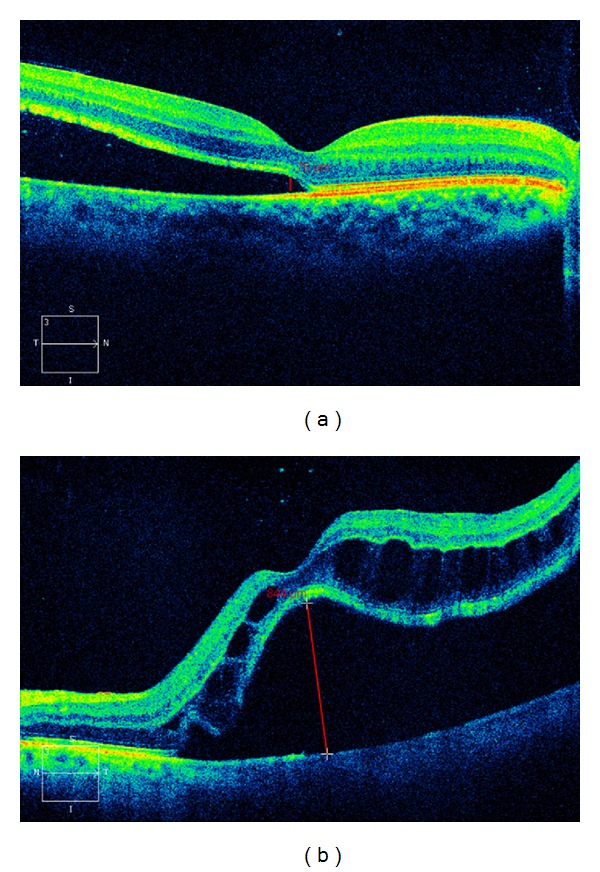
Pre-operative optical coherence tomography (OCT) findings for normal retinal architecture and edema of the outer nuclear later of the retina. (a) A 26-year-old female showing normal retinal architecture. Her preoperative logMAR visual acuity was 0.07, and duration of retinal detachment was 9 days. (b) A 52-year-old female showing edema of the outer nuclear layer of the retina. Her preoperative logMAR visual acuity 1.15 and duration of retinal detachment were 52 days.

The subjects were divided into 2 groups based on the preoperative retinal architecture, that is, Group 1 with a normal retinal architecture and Group 2 with edema affecting the outer nuclear layer of the retina. The mean age of the subjects was 35 ± 12.7 years in Group 1 and 56 ± 12.1 years in Group 2. The mean preoperative logMAR visual acuity was 0.17 ± 0.17 in Group 1 and 1.08 ± 0.71 in Group 2, while the mean logMAR visual acuity 6 months after surgery was 0.06 ± 0.20 for Group 1 and 0.44 ± 0.45 for Group 2. The mean age, preoperative visual acuity, and visual acuity 6 months after surgery showed significant differences between Group 1 and Group 2 (*P* < 0.01). (Table 2).  Figure 2 shows typical examples for these groups.

### 3.5. Relationship of Postoperative Visual Acuity to Postoperative IS/OS Junction Continuity

**Figure 3:**
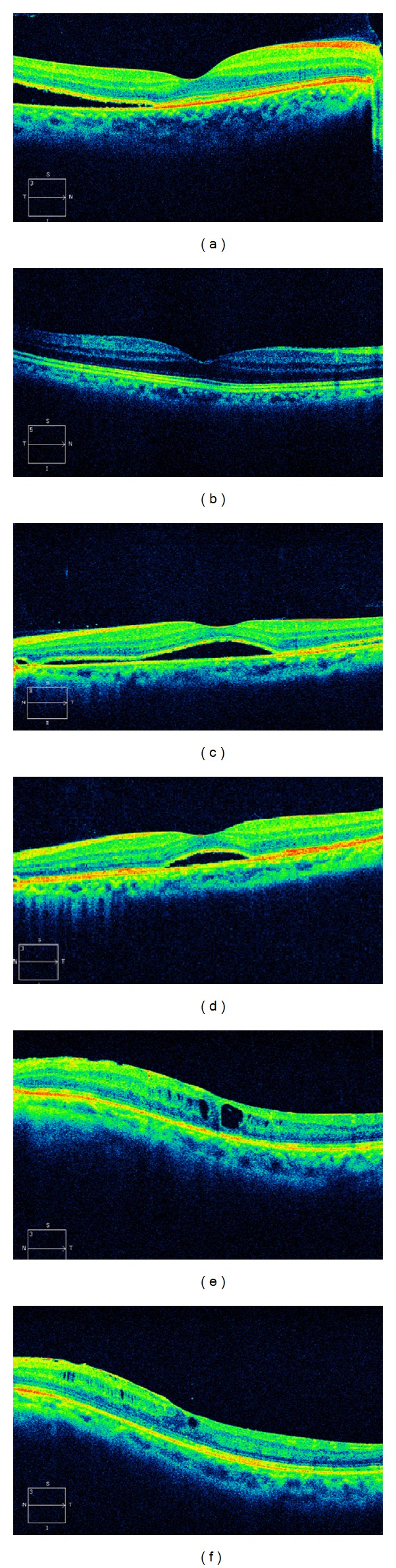
Postoperative optical coherence tomography (OCT) findings for presence/absence of IS/OS junction continuity. A 26-year-old female with the presence of IS/OS junction continuity both 3 months (a) and 6 months (b) after scleral buckling surgery. LogMAR visual acuity was −0.08 prior to the surgery, 0.00 3 months after surgery, and −0.08 6 months after surgery in this eye. A 26-year-old male with the absence of IS/OS junction continuity 3 months (c) and presence of IS/OS junction continuity 6 months (d) after scleral buckling surgery. LogMAR visual acuity was 0.30 prior to the surgery, 0.15 3 months after surgery, and 0.09 6 months after surgery in this eye. A 49-year-old male with the absence of IS/OS junction continuity both 3 months (e) and 6 months (f) after vitreous surgery. LogMAR visual acuity was 2.00 prior to the surgery, 1.09 3 months after surgery, and 1.00 6 months after surgery in this eye.

Table 3 shows the relationship between the presence of IS/OS junction continuity 3 and 6 months after surgery and the visual acuity 6 months after surgery, while Figure 3 displays typical examples. The visual acuity 6 months postoperatively was 0.07 ± 0.14 for 11 eyes that showed IS/OS junction continuity both 3 and 6 months after surgery, while it was 0.12 ± 0.11 for 5 eyes in which IS/OS junction continuity was not noted 3 months after surgery but was observed at 6 months. The visual acuity 6 months postoperatively was 0.77 ± 0.42 for 9 eyes without IS/OS junction continuity both 3 and 6 months after surgery; that is, postoperative visual acuity was significantly worse than that of eyes with IS/OS junction continuity both 3 and 6 months after surgery (*P* < 0.01).

### 3.6. Relationship of Postoperative Visual Acuity to Postoperative IS/OS Junction and ELM Continuity

**Figure 4:**
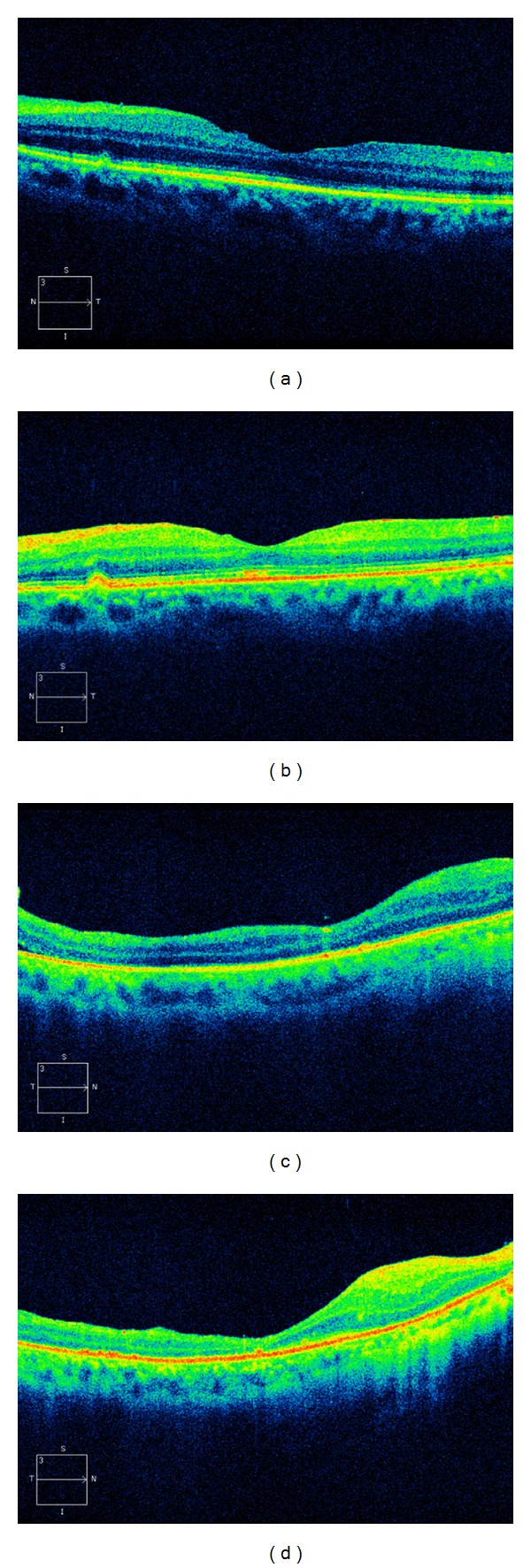
Postoperative optical coherence tomography (OCT) findings for presence/absence of IS/OS junction and external limiting membrane (ELM) continuity. A 58-year-old male with the presence of IS/OS junction continuity and absence of ELM continuity 3 months (a) and 6 months (b) after vitreous surgery. LogMAR visual acuity was 0.69 prior to the surgery, 0.15 3 months after surgery, and 0 6 months after surgery in this eye. A 54-year-old male with the absence of both IS/OS junction and ELM continuity 3 months (c) and 6 months (d) after vitreous surgery. LogMAR visual acuity was 2.0 prior to the surgery, 1.52 3 months after surgery, and 1.52 6 months after surgery in this eye.

Table 4 shows the IS/OS junction and ELM continuity 3 and 6 months postoperatively, as well as the mean logMAR visual acuity 6 months after surgery. Eyes without IS/OS junction continuity 3 months after surgery were classified into 2 groups based on the presence of ELM continuity. The first group consisted of with ELM continuity and the second group without ELM continuity. IS/OS junction continuity was noted 6 months after surgery in 4 of 6 eyes with ELM continuity (67%), and the logMAR visual acuity was 0.22 ± 0.25 6 months postoperatively. In contrast, IS/OS junction continuity was noted 6 months after surgery in only 1 of 9 eyes without ELM continuity (12%), and the logMAR visual acuity was 0.88 ± 0.36 6 months postoperatively. The visual outcome 6 months postoperatively was more favorable for the eyes with ELM continuity 3 months after surgery than for the eyes without it (*P* < 0.01).  Figure 4 shows typical examples for these groups.

## 4. Discussion

OCT has made it possible to estimate the visual outcome of retinal detachment surgery by analyzing postoperative morphologic changes of the photoreceptor cell layer of the retina [1]. The preoperative visual acuity [2], duration of detachment [3], height of macular detachment [4, 5], and presence/absence of vitreous traction [6] have been reported as preoperative factors related to restoration of macular function after surgery for retinal detachment, while cystoid macular edema [3, 7], epimacular membrane [3], retinal wrinkles [5, 8], shifting of the retinal pigment epithelium [8], and subretinal fluid [5] have been reported as postoperative factors.

 It has been reported that recovery of visual function is possible when surgery is done within 1 week after the onset of retinal detachment [9]. However, the logMAR visual acuity 6 months after surgery showed no significant difference between our groups with an interval from onset to surgery before 10 days and 10 days and more.

 The incidence of postoperative residual retinal detachment affecting the macular region varies among surgical techniques. According to Kambara et al. [10], it is 63% after transscleral surgery and only 13% after vitrectomy. In our series, postoperative subretinal fluid was noted in 86% after scleral buckling versus 33% after vitrectomy, so the incidence of postoperative residual subretinal fluid was higher after scleral buckling. Chihara and Nao-i [11] investigated subretinal fluid in 10 patients with fresh retinal detachment and reported that the fluid was absorbed within 23 hours if the lacuna was closed. Postoperative subretinal fluid may be ascribable to disturbance of the choroidal circulation, but its cause is unknown. It has been reported by many researchers that prolonged retention of subretinal fluid is one of the causes of delayed restoration of visual acuity [5, 12]. In this study, the visual acuity 6 months after surgery was also worse in the patients who had residual subretinal fluid for 3 months or more after surgery. According to Hagimura et al. [5], the visual outcome becomes worse as the height of retinal detachment increases. We obtained similar results; that is, the preoperative and postoperative visual acuity of eyes with a preoperative HRD ≥ 1,000 *μ*m was worse than the preoperative and 6-month postoperative visual acuity of eyes with a preoperative HRD < 1,000 *μ*m. It has been reported that structural abnormalities caused by retinal detachment are related to the visual outcome, and if there is retinal separation or a wavy external layer before surgery, the final visual acuity is unfavorable [5]. In our series, the visual acuity 6 months after surgery was worse for the eyes with preoperative edema of the outer nuclear layer of the retina than for the eyes with a normal retinal architecture before surgery. 

 The following reports have been published concerning the photoreceptor cell layer. Schocket et al. [13] reported that IS/OS junction disruption was noted in the reattached retina, while Wakabayashi et al. [14] stated that the postoperative visual acuity of eyes with macular detachment was significantly correlated with IS/OS junction and ELM defects. Because the ELM is the boundary between the external nuclear layer, which contains the nuclei of the photoreceptor cells, and the inner segment of the photoreceptor cell layer, it is suggested that ELM and IS/OS junction defects may also influence the nuclei of the photoreceptor cells. This is supported by the report that apoptosis of photoreceptor cells and separation of the outer segment occur after experimental retinal detachment, while the outer segment regenerates after retinal reattachment [15]. Kawashima et al. [16] suggested that the improvement of vision after reattachment of the retina was correlated with a decrease of IS/OS junction disruption and that resolution of ELM disruption was a prerequisite for restoration of the IS/OS junction. In our series, the visual acuity at 6 months postoperatively was better if there was IS/OS junction continuity at both 3 and 6 months after surgery than if this was not the case. In addition, the rate of achieving IS/OS junction continuity was higher, and the visual acuity 6 months postoperatively was better for the eyes with ELM continuity 3 months after surgery. 

 In patients with macula-off retinal detachment, OCT makes it possible to evaluate the preoperative retinal architecture and HRD, as well as the postoperative IS/OS junction and ELM. Thus, OCT may be useful for estimating the visual outcome after surgery in such patients.

## Figures and Tables

**Table 1:** Pre- and postoperative logMAR visual acuity compared with the duration of retinal detachment.

	Number of eyes	Mean age (years old)	LogMAR visual acuity	
			Before operation	6 months postoperatively
HRD ≥ 1000 *μ*m	7	58.0 ± 11.8	1.54 ± 0.50*	0.70 ± 0.36*
HRD < 1000 *μ*m	19	49.0 ± 16.3	0.65 ± 0.67*	0.25 ± 0.38*

*P < 0.05 (Mann-Whitney *U* test).

**Table 2:** Comparison of the mean logMAR visual acuity in eyes with normal retinal architecture, Group 1, and eyes with edema of the outer nuclear layer of the retina, Group 2, in the pre- and postoperative logMAR visual acuity.

Group	Number of eyes	Duration of retinal detachment (days)	Mean age (years old)	Mean logMAR visual acuity	
				Before operation	6 months postoperatively
1	7	14 ± 13	35 ± 12.7**	0.17 ± 0.17**	0.06 ± 0.20**
2	19	25 ± 29	56 ± 12.1**	1.08 ± 0.71**	0.44 ± 0.45**

**P < 0.01 (Mann-Whitney *U* test).

**Table 3:** Relationship of postoperative visual acuity to postoperative IS/OS junction continuity.

IS/OS junction continuity		Number of eyes	Postoperative logMAR visual acuity 6 months postoperatively
3 months postoperatively	6 months postoperatively		
+	+	11	0.07 ± 0.14
−	+	5	0.12 ± 0.11**
−	−	9	0.77 ± 0.42**

**P < 0.01 (Mann-Whitney *U* test).

**Table 4:** Relationship of postoperative visual acuity to postoperative IS/OS junction and ELM continuity.

3 months postoperatively		6 months postoperatively	Number of eyes	Postoperative logMAR visual acuity
IS/OS	ELM	IS/OS		
−	+	+	4	0.22 ± 0.25**
		−	2	
−	−	+	1	0.88 ± 0.36**
		−	8	

**P < 0.01 (Mann-Whitney *U* test).
